# Sex-dependent effects of the uncompetitive N-methyl-D-aspartate receptor antagonist REL-1017 in G93A-SOD1 amyotrophic lateral sclerosis mice

**DOI:** 10.3389/fneur.2024.1384829

**Published:** 2024-05-03

**Authors:** Martina Colognesi, Atea Shkodra, Daniela Gabbia, Hibiki Kawamata, Paolo L. Manfredi, Giovanni Manfredi, Sara De Martin

**Affiliations:** ^1^Department of Pharmaceutical and Pharmacological Sciences, University of Padova, Padova, Italy; ^2^Brain and Mind Research Institute, Weill Cornell Medicine, New York, NY, United States; ^3^Relmada Therapeutics, Coral Gables, FL, United States

**Keywords:** ALS, REL-1017, esmethadone, NMDAR antagonism, neuronal plasticity, oxidative stress

## Abstract

**Introduction:**

The pathogenesis of amyotrophic lateral sclerosis (ALS), a fatal neurodegenerative disease caused by the demise of motor neurons has been linked to excitotoxicity caused by excessive calcium influx via N-methyl-D-aspartate receptors (NMDARs), suggesting that uncompetitive NMDAR antagonism could be a strategy to attenuate motor neuron degeneration. REL-1017, the dextro-isomer of racemic methadone, is a low-affinity uncompetitive NMDAR antagonist. Importantly, in humans REL-1017 has shown excellent tolerability in clinical trials for major depression.

**Methods:**

Here, we tested if REL-1017 improves the disease phenotypes in the G93A SOD1 mouse, a well-established model of familial ALS, by examining survival and motor functions, as well as the expression of genes and proteins involved in neuroplasticity.

**Results:**

We found a sex-dependent effect of REL-1017 in G93A SOD1 mice. A delay of ALS symptom onset, assessed as 10%-decrease of body weight (*p* < 0.01 vs. control untreated mice) and an extension of lifespan (*p* < 0.001 vs. control untreated mice) was observed in male G93A SOD1 mice. Female G93A SOD1 mice treated with REL-1017 showed an improvement of muscle strength (*p* < 0.01 vs. control untreated mice). Both males and females treated with REL-1017 showed a decrease in hind limb clasping. Sex-dependent effects of REL-1017 were also detected in molecular markers of neuronal plasticity (PSD95 and SYN1) in the spinal cord and in the GluN1 NMDAR subunit in quadricep muscles.

**Conclusion:**

In conclusion, this study provides preclinical *in vivo* evidence supporting the clinical evaluation of REL-1017 in ALS.

## 1 Introduction

Amyotrophic lateral sclerosis (ALS) is a fatal neurodegenerative disease caused by the demise of motor neurons. Globally, the estimated prevalence of ALS cases in 2020 was 121,028 (1). Approximately 90% of ALS cases have no family history (sporadic ALS), while 10% are due to inherited mutations.

Numerous lines of evidence indicate the involvement of excitotoxicity in the pathogenesis of ALS (2), suggesting that reducing excessive Ca^2+^ influx via NMDA receptors could postpone or prevent motor neuronal death. Excitotoxicity can be induced by excessive synaptic glutamate, due to either increased release of glutamate or impaired glutamate uptake; excitotoxicity can also be induced by endogenous or exhogenous molecules acting as competitive agonists at the glutamate site or molecules acting as positive allosteric modulators of the NMDARs. Excessive stimulation of NMDARs can cause intracellular excessive Ca^2+^ influx and neuronal dysfunction and death. A role for the dysregulation of glutamate homeostasis in ALS-mediated neurodegeneration has long been established by *in vitro* and *in vivo* studies, which demonstrated that motor neurons are markedly vulnerable to glutamate excitotoxicity (3, 4). However, while studies have often investigated the role of α-amino-3-hydroxy-5-methyl-4-isoxazole propionic acid receptors (AMPARs) (5–7), they have rarely focused on NMDARs.

In the CNS, NMDARs are expressed in both neuronal and non-neuronal cells; Ca^2+^ influx via NMDARs regulates numerous functions, including neurotransmission, neuronal development, and plasticity, and also participate in neurodegenerative processes (8, 9). Moreover, drugs antagonizing NMDAR activity have been recently approved for neurodegenerative and neuropsychiatric diseases (e.g., memantine for AD and ketamine for depression). Therefore, the NMDAR could be considered a potential therapeutic target for ALS.

REL-1017 (esmethadone), the dextro-isomer of racemic methadone, is a low-affinity uncompetitive NMDAR antagonist which blocks channels co-expressing NMDAR1 and NMDAR2 subunits with a preference for GluN2D subtypes (10, 11), REL-1017 has a 20–40 fold lower affinity for the mu opioid receptor compared to the levo-isomer (12, 13) and no clinically meaningful opioid agonist effects (14–17). REL-1017 has mTOR- and brain derived neurothrophic factor (BDNF)-dependent synaptogenic properties in cultured neurons and stimulates dendritic spine growth *in vivo* (13, 18). Interestingly, REL-1017 appears to modulate BDNF (18, 19) a neurotrophin with pleiotropic functions, including neural development and plasticity, as well as oxidative stress responses (20). This finding could be relevant to ALS, since numerous studies have reported increased oxidative stress in ALS *postmortem* tissues, particularly in the spinal cord and motor cortex (21, 22). Taken together, this evidence suggests that the unique pharmacological properties of REL-1017 could be beneficial in ALS.

Mutations in superoxide dismutase 1 (SOD1) account for approximately 20% of familial ALS cases and 2% of total cases (23) and nearly 150 mutations in SOD1 have been associated with inherited ALS (24). The antisense oligonucleotide Tofersen targeting SOD1 mRNA has recently been approved in the USA for the therapy of ALS in adults carrying SOD1 mutations. Although the Toferesen clinical trial showed a decrease of SOD1 and NfL, a biomarker of neuronal degeneration, there were no measurable clinical improvements (25), suggesting that additional therapeutic measures may need to be implemented in people with SOD1 ALS. Transgenic mice expressing high levels of G93A mutant human SOD1 gene (G93A SOD1) manifest very aggressive motor neuron degeneration, which leads to paralysis and early death (26, 27); therefore, this mouse model recapitulates key features of ALS. SOD1 mutant mice show hallmarks of excitotoxicity, neuroinflammation, and oxidative stress (28–31), suggesting that NMDAR modulation by REL-1017 could represent a potential therapeutic strategy in this mouse model of SOD1 ALS.

In this study, to obtain *in vivo* preclinical evidence on the potential use of REL-1017 for ALS treatment, we tested REL-1017 efficacy in the G93A SOD1 mouse by evaluating its effects on survival and motor phenotype, as well as on the expression of genes involved in neuroplasticity.

## 2 Materials and methods

### 2.1 *In vivo* studies

All experimental protocols involving animals were authorized by the Animal Care and Use Ethics Committee of the University of Padova (OPBA) and the Italian Ministry of Health (Auth No. 681/2019-PR). G93A SOD1 female and male mice (*n* = 8 per group) were purchased from the Jackson Laboratories [B6SJL-Tg (SOD1 × G93A)1Gur/J] and treated with subcutaneous daily injections of different doses of REL-1017, starting at 45 days of age, before the onset of symptoms (26, 32). A control group for each sex (*n* = 8) was treated daily with saline (vehicle). Male mice were treated either with 3 or 6 mg/kg of REL-1017, whereas females were treated with 10 or 20 mg/kg. Lower doses in males were used because chronic treatment with REL-1017 s.c. at 20 mg/kg/day can cause skin alterations in male mice (data not shown). The treatment was performed every day until sacrifice.

### 2.2 Motor function analyses

Motor function analyses were performed weekly, starting at 60 days of age. Prior to each test, animals underwent one week of training.

For the grip test, a horizontal dynamometer (Bioseb BIO-GS3; Vitrolles, France) was used to assess neuromuscular function as maximal peak of strength of the animal forelimbs. Animals were allowed to grab the dynamometer and then gently pulled by tail with a constant strength to measure forelimb grip strength.

In the clasping test, the animals were lifted by the tail and the hindlimb position was observed for 10 s. A score was assigned to the degree of hindlimb splay (0 to 3), where 0 is absence of hindlimb clasping and 3 is maximum splay for 5 s or more.

In the rotarod test, performed for the assessment of motor coordination and balance, mice were placed onto the rotating rod (Ugo Basile, Milano, Italy) at constant speed of 28 rpm for a maximum of 5 min, and the latency to fall from the rod was measured.

### 2.3 Body weight

The body weight of males and females was weekly assessed starting from 45 days of age till the experimental endpoint.

### 2.4 Tremor

The severe progressive denervation of the hindlimb muscles reported in G93A SOD1 mice is known to be associated with the onset of mild and severe tremors (33). Female and male mice were daily monitored to establish the first day from the onset of severe tremors.

### 2.5 Survival criteria

The disease progression in G93A SOD1 mice is associated with many debilitating symptoms, ranging from body weight decrease to motor dysfunctions of hindlimbs and consequent paralysis due to motorneuron death (34). To evaluate any influence of pharmacological treatment on mice worsening of ALS-like symptoms, the experimental endpoint was reached when the animals were completely unable to right themselves after being placed on their side.

### 2.6 *Ex vivo* studies

#### 2.6.1 Total RNA extraction from spinal cord and quadriceps

Total RNA was extracted from spinal cord and quadriceps after homogenization and purified with the SV Total RNA Isolation System (Promega Corporation, Madison, WI, United States), as described previously (35). Spinal cord tissue was homogenized in lysis buffer and mRNA was purified by means of silica-gel-based columns, according to the manufacturer’s instructions. A DNAse treatment was used during RNA extraction to prevent genomic DNA contamination. Purified RNA was eluted in a final volume of 100 μL RNAse-free water. Aliquots were stored at −80°C until use.

#### 2.6.2 qRT-PCR analyses

qRT-PCR was carried out by using the commercial One Step SYBR PrimeScript RT-PCR Kit (Takara, Mountain View, CA, United States) (36). The RNA was amplified and quantified by qRT-PCR. The samples were diluted to obtain 180 ng per wells, and then 1.8 μL of samples were added in a 48-well microplate for PCR together with 3.2 μL of Mastermix, containing 2X One Step TB Green RT-PCR Buffer III, PrimeScript RT enzyme Mix II, and ROX Reference Dye II (One Step SYBR PrimeScript RT-PCR kit II; Takara; Japan).

The qRT-PCR thermal program was as follows: 15 min at 50°C and 2 min at 95°C for the reverse transcription, 40 cycles of 15 s at 95°C and 60 s at 60°C for the PCR reaction, and then 15 s at 95°C, 15 s at 55°C and 15 s at 95°C for the dissociation curve. During the exponential phase, the fluorescence signal threshold was calculated, and the cycle threshold (Ct) was ascertained. The Ct values were used to calculate the relative mRNA expression, according to the Pfaffl mathematical quantification method (37). All samples were tested in triplicate and β-actin mRNA was used for normalization.

**Table 1:** 

Gene	Forward primer (5′-3′)	Reverse primer (5′-3′)
GluN1	TGCGCGTCTACAACTGGAAC	CATAGGACAGTTGGTCGAGGT
SYN1	TTGTGGCTCATGCCAATGGT	TCCATTACGTGCCATGCTGA
PSD95	TACCAAGATGAAGACACGCCC	TTCCGTTCACCTGCAACTCAT
β-actin	ATGTGGATCAGCAAGCAGGA	AAGGGTGTAAAACGCAGCTCA

#### 2.6.3 Total protein extraction from quadriceps and western blot analyses

Protein lysates were obtained from quadriceps after homogenization with IKA T-25 digital Ultra-Turrax in modified RIPA buffer with the addition of a cocktail of protease inhibitors (cOmplete Mini, Roche Diagnostics GmbH, Mannheim, Germany). Aliquots were stored at −80°C until use. The levels of GluN1 subunit of NMDA receptor were estimated by western blot of 30 μg of total protein (anti-GluN1 antibody diluted 1:1000, ab68144; Abcam, Cambridge, United Kingdom). The signal intensity of the immunoreactive bands was analyzed with the Quantity One software (Bio-Rad Laboratories; Hercules, CA, United States) and normalized to that of the GAPDH, as previously described (38).

### 2.7 Statistical analyses

Statistical analyses were performed by GraphPad Prism software, ver. 10.0.3 (GraphPad Software Inc., San Diego, CA, United States). Data were compared by one-way or two-way ANOVA, as appropriate. ANOVA was followed up by the Dunnett’s or Tukey’s *post-hoc* tests. A *p*-value of *p* < 0.05 was considered statistically significant. Unless otherwise stated, data are expressed as means ± S.E.M.

## 3 Results

### 3.1 REL-1017 effect on body weight and lifespan

Only one male mouse had adverse skin effects to REL-1017 at the dose of 6 mg/kg, showing hair loss and dermal ulceration that required topical antibiotic treatment.

**Figure 1:**
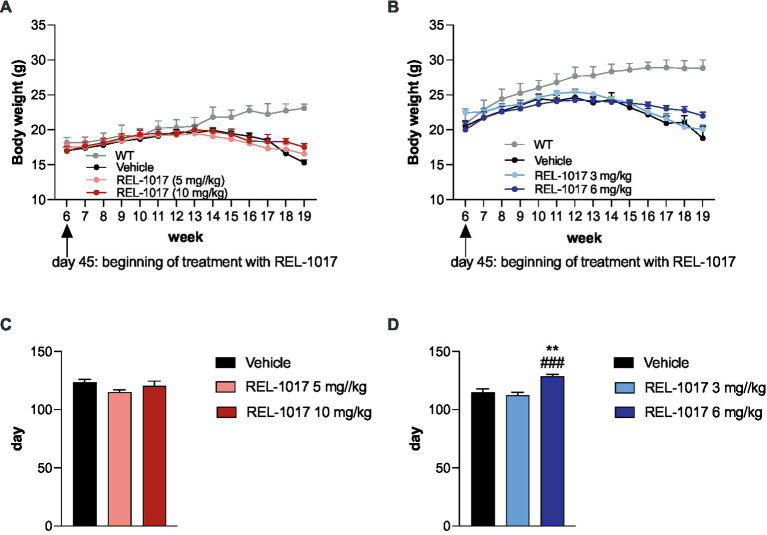
Effects of daily REL-1017 s.c. chronic treatment on body weight. The graphs show body weights of the wild type (gray line), and of G93A SOD1 mice, starting from day 45 (beginning of REL-1017 treatment) for female **(A)** and male **(B)** mice. Data are shown as mean ± SEM for each time point. Onset of 10%-body weight loss in female **(C)** and male **(D)** G93A SOD1 mice. ^**^p < 0.01 vs. vehicle, ^###^*p* < 0.001 vs. mice treated with REL-1017 3 mg/kg.

In G93A SOD1 mice, the onset of ALS symptoms was assessed by the appearance of severe tremor and body weight loss. We considered a 10% loss of body weight as the beginning of symptoms. In G93A SOD1 mice, REL-1017 treatment had no effect on the onset of tremor (Supplementary Figure S1), which we considered as the first motor ALS symptom. G93A SOD1 mice had significantly lower body weight compared to wild-type mice (*p* < 0.01 for the interaction time X genotype calculated with two-way ANOVA) from week 14 in females (Figure 1A) and from week 12 in males (Figure 1B). The body weight of wild-type mice was not affected by REL-1017 administration (data not shown). In both sexes, we observed a trend for attenuation of the body weight loss in G93A SOD1 mice treated with the highest doses of REL-1017 (10 mg/kg for females, 6 mg/kg for males), but this difference was not statistically significant. However, a significant increase of the time needed to lose 10% of maximum body weight was observed in G93A SOD1 males with the highest dose of REL-1017 (Figure 1C). This effect was not observed in female mice (Figure 1D).

**Figure 2:**
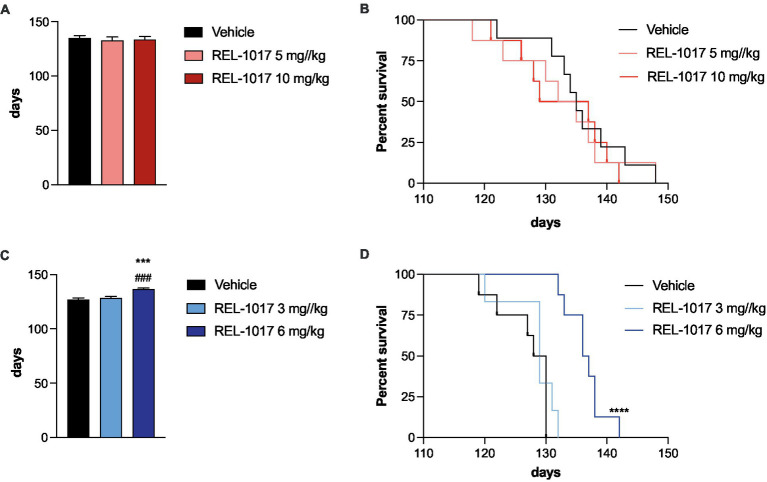
Effect of REL-1017 on survival of G93A SOD1 mice. Lifespan (**A** for females, **C** for males) and Kaplan–Meier survival curves (**B** for females, **D** for males) of G93A SOD1 mice. ^***^p < 0.001 vs. vehicle, ^###^*p* < 0.001 vs. mice treated with REL-1017 3 mg/kg. In **(D)** ^****^p < 0.0001 vs. vehicle and REL-1017 3 mg/kg.

A small but significant increase of lifespan was observed in G93A SOD1 male mice treated with 6 mg/kg REL-1017. The Mantel_Cox log-rank test indicated a significant difference between survival curves (*p* < 0.0001), with median survivals of 129 days in vehicle-treated and 3 mg/kg-treated male mice and of 134.5 days in mice treated with 6 mg/kg (Figures 2C,D). This effect was not observed in females, where no significant difference could be demonstrated between survival curves, with a median survival of 135 days for vehicle-treated female mice and 133.5 days for REL-1017 treated mice with both tested doses (Figures 2A,B).

### 3.2 REL-1017 effects on motor function

**Figure 3:**
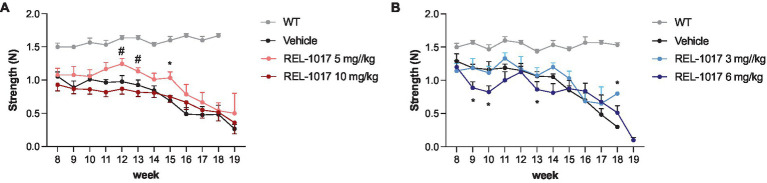
Grip strength test. The graphs show the effect of time and treatment on the grip strength for female **(A)** and male **(B)** mice. ^*^p < 0.05 vs. vehicle, ^#^*p* < 0.05 vs. REL-1017 10 mg/kg by two-way ANOVA with correction.

Disease progression in G93A SOD1 mice is associated with a decline of muscle strength and coordination. High-dose treatment with REL-1017 did not improve forelimb grip strength in G93A SOD1 mice compared to vehicle. However, 5 mg/kg/day REL-1017 improved grip strength in females (Figure 3A). No beneficial effects were observed in G93A SOD1 males (Figure 3B).

**Figure 4:**
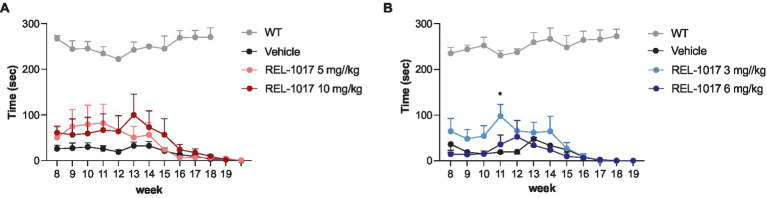
Rotarod test. The graphs show the effect of time and treatment on the rotarod test for female **(A)** and male **(B)** mice. ^*^p < 0.05 vs. vehicle, two-way ANOVA with correction.

In our hands, G93A SOD1 mice showed a significantly worse rotarod performance compared to wild-type mice as early as 8 weeks of age. Female mice treated with 10 mg/kg REL-1017 tended to improve their performance with respect to vehicle-treated females (Figure 4A), although the statistical significance could not be reached, and male mice treated with 3 mg/kg REL-1017 preformed better than vehicle-treated mice (Figure 4B).

**Figure 5:**
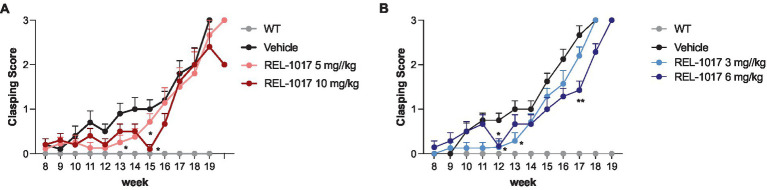
Clasping test. Effect of time and treatment on the clasping score for female **(A)** and male **(B)** mice. ^*^p < 0.05 vs. vehicle, by two-way ANOVA with correction.

G93A SOD1 mice showed typical clasping by 14 weeks of age (*p* < 0.0001), and REL-1017 treatment at the highest dose led to a moderate reduction of clasping in mutant mice (Figures 5A,B).

### 3.3 REL-1017 modulation of neuroplasticity gene PSD95 in spinal cord

**Figure 6:**
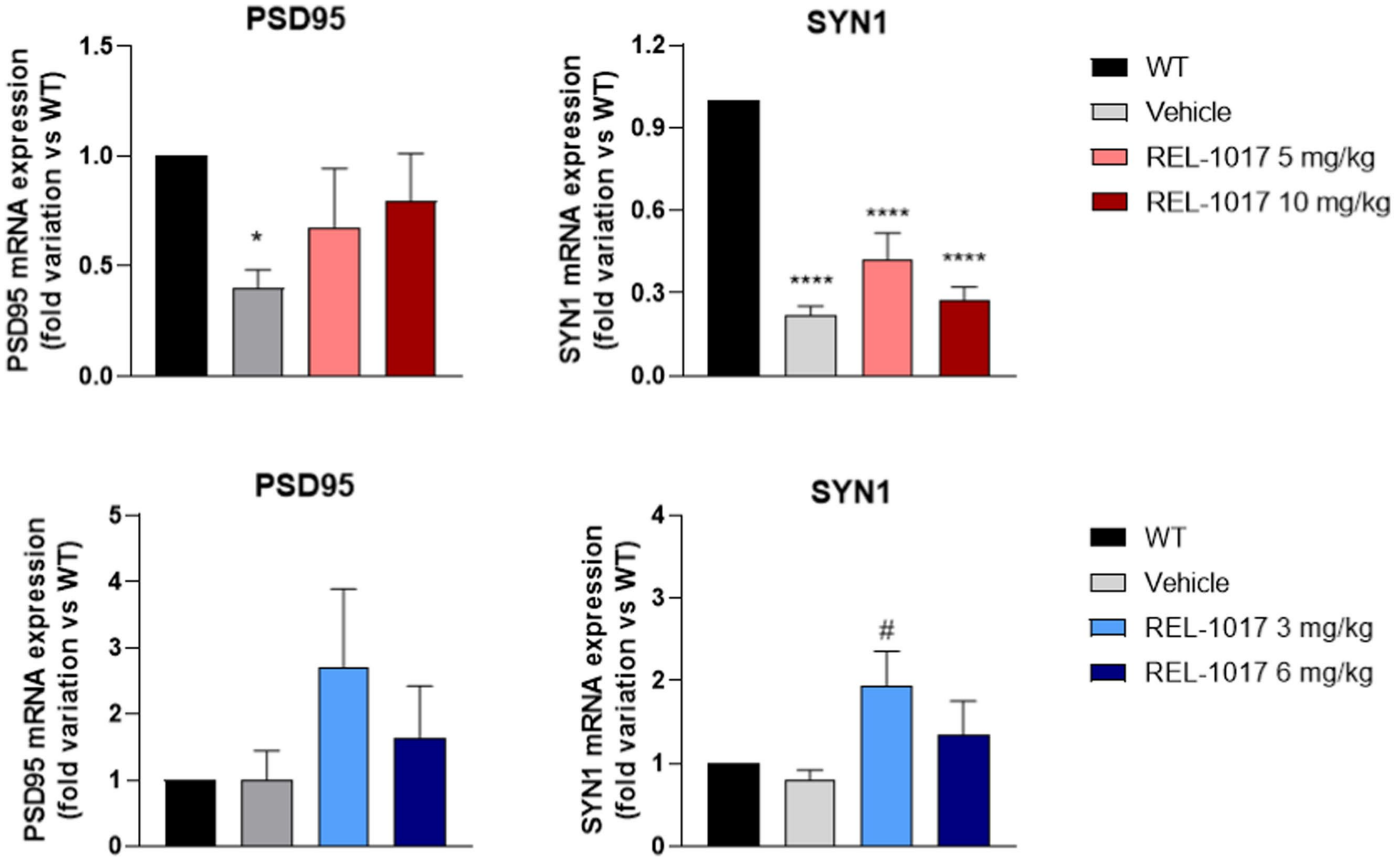
Effect of REL-1017 treatment on mRNA expression in spinal cord of two genes involved in neuroplasticity. mRNA expression of PSD95 in females **(A)** and males **(C)**, SYN1 (panels **B** and **D**, females and males repectively), Data are presented as mean ± S.E.M. ^*^p < 0.05, ^****^p < 0.0001 vs. WT mice, ^#^*p* < 0.05 vs. vehicle, by one-way ANOVA with correction (*n* = 6).

Mice were euthanized when they were unable to right themselves after being placed on their side. Spinal cords were dissected for mRNA and protein studies of neuroplasticity genes. G93A SOD1 female mice showed a significant decrease of the PSD95 (Figure 6A) and SYN1 (Figure 6B) mRNA expression with respect to wild-type female mice. REL-1017 treatment counteracted the decrease of PSD95 in a dose-dependent manner, while it had no effect on the decrease observed in SYN1 mRNA. Interestingly, 3 mg/kg REL-1017 increased PSD95 and SYN1 mRNA levels in male G93A SOD1 relative to vehicle treated G93A SOD1 males (Figures 6C,D).

### 3.4 REL-1017 modulation of GluN1 mRNA and protein expression in quadriceps

**Figure 7:**
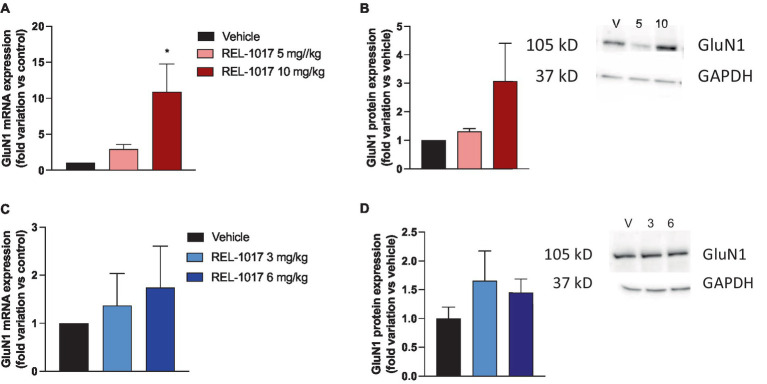
Effect of REL-1017 treatment on mRNA **(A,C)** and protein **(B,D)** of GluN1 subunit in muscle of G93A SOD1 mice. Data are presented as mean ± S.E.M. ^*^p < 0.05 vs. vehicle-treated mice (*n* = 3 mice per group). Representative images of the immunoreactive bands are reported in panels **B** and **D**. V, vehicle; 5, REL-1017 5 mg/kg; 10, REL-1017 10 mg/kg; 3, REL-1017 3 mg/kg; 6, REL-1017 6 mg/kg.

Quadricep muscle was dissected for protein analyses. Since glutamate released at the neuromuscular junction can exert its effects by activating the postsynaptic NMDAR containing the GluN1 subunit expressed in the postsynaptic membranes of skeletal muscles (39), and we previously observed that REL-1017 treatment can modulate NMDAR expression, we evaluated GluN1 expression in quadriceps. In female G93A SOD1 mice, an increase in GluN1 expression at the mRNA and protein levels was observed with the highest doses of REL-1017 (Figures 7A,B), whereas no significant effect in male mice was exerted by the treatment (Figures 7C,D).

### 3.5 REL-1017 modulation of NOX4 mRNA expression in quadriceps

**Figure 8:**
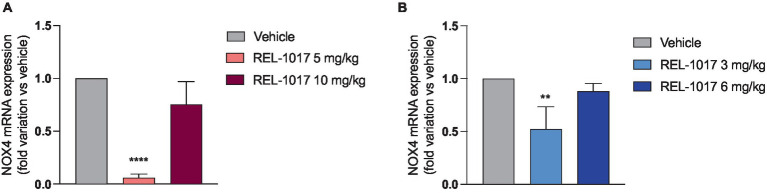
Effect of REL-1017 treatment on mRNA of NOX4 subunit in muscle of female **(A)** and male **(B)** G93A SOD1 mice. Data are presented as mean ± S.E.M. ^**^p < 0.01, ^****^p < 0.0001 vs. vehicle-treated mice (*n* = 5 mice per group).

Since NMDAR excessive activation by glutamate can result in an oxidative stress increase due to the NADPH-oxidase isoform 4 (NOX4), which produces oxygen peroxide and consequrntly acrive oxygen species (40), we evaluated NOX4 expression in quadriceps. In both female and male G93A SOD1 mice, NOX4 mRNA expression was significantly reduced by the treatment with the lowest dose of REL-1017 (Figures 8A,B).

## 4 Discussion

ALS is a rapidly progressing and debilitating neurodegenerative disease, characterized by loss of upper and lower motor neurons, leading to death. Although an increasing incidence of ALS has been reported worldwide (41), to date only few therapeutic approaches are available, including the AMPAR modulator Riluzole, the antioxidant Edaravone, and the combination of tauroursodeoxycholic acid and sodium phenylbutyrate (AMX0035) (42).

Much evidence suggests a pivotal role for NMDAR-mediated excitotoxicity in the pathogenesis of ALS (2, 3); for this reason we investigated the *in vivo* effect of the NMDA receptor antagonist REL-1017 on ALS onset and progression in the G93A SOD1 mouse.

Body weight assessment, first symptoms onset, as well as motor tests were used as parameters to evaluate the *in vivo* progression of ALS. Wild-type mice, treated either with vehicle or with high-dose of REL-1017 (10 mg/kg and 20 mg/kg), did not show any significant effects on body weight and motor performance (data not shown). On the other hand, we found a moderate dose-dependent trend indicating a protective effect on body weight loss in the last weeks of life in the G93A SOD1 mice treated with REL-1017. Considering that body weight decrease is regarded as prognosis factor for shorter survival of patients with ALS (43, 44), we decided to evaluate the 10% loss of body weight relative to the maximum weight reached, and we detected a significant increase of the time required for the onset of this symptom in male mice treated with 6 mg/kg/day REL-1017. The results correlated with an increase of survival in male mice. Another important parameter associated with ALS phenotype is the progressive loss of motor function. REL-1017 treatment delayed hindlimbs clasping reflex in male mice, whereas G93A SOD1 females treated with the highest dose improved significantly both forelimb the grip strength and the clasping score compared to placebo, indicating an effect of REL-1017 on motor function. We observed a trend towards improvement of rotarod performance, although not statistically significant in female mice treated with the highest REL-1017 dose and in male mice treated with the lowest REL-1017 dose.

Next, we focused on the effect of REL-1017 on molecular markers of ALS in spinal cord and skeletal muscle, as these are the main tissues targeted by ALS pathology (45). Considering the previously reported modulation of neuroplasticity after treatment with REL-1017 (13, 18, 19, 46), and the importance of neuroplasticity in ALS (47, 48) we evaluated the mRNA expression of PSD95 and SYN1 in end-stage spinal cord, two synaptic plasticity genes known to be altered in ALS (49). We observed a significant decrease of PSD95 and SYN1 mRNA levels in G93A SOD1 female mice. Interestingly, the decrease of PSD95 expression was prevented by REL-1017 in a dose-dependent manner. In male G93A SOD1 mice, we observed a trend for increase of these mRNAs in mice treated with the lowest dose, suggesting a modulatory role of REL-1017.

Finally, we evaluated the effect of REL-1017 on mRNA and protein levels of the GluN1 subunit of NMDA receptor in quadricep, which is needed for a functional channel. The highest dose of REL-1017 caused a significant increase of GluN1 expression in G93A SOD1 female mice. Although further studies will be needed to assess the role of NMDAR in skeletal muscles and the effect of REL-1017, increased NMDAR expression in specific brain areas was shown to boost neuroplasticity (50), and this putative mechanism has been suggested also as a neuroprotective effect of REL-1017 (19, 51).

An intriguing observation in this study is the potential effect of REL-1017 and NMDAR antagonist in general on reduction of oxidative stress, as suggested by decreased expression of Nox4, which is known to be involved in oxidative stress processes linked to NMDAR activation (40). Riluzole is an inhibitor of glutamate release with antioxidant properties found to be effective for ALS. Interestingly, Rammes et al. (52) suggested the combined use of riluzole with NMDAR uncompetitive antagonists, to potentially gain an additive or synergistic effect from the use of the two different NMDAR modulating drugs in ALS treatment. Therefore, it could be hypothesized that NMDAR antagonists may exert protective effects in G93A SOD1 mice by reducing oxidative stress, although further studies are needed to test this hypothesis.

In summary, in this study we report sex-dependent effects of REL-1017 in a well-established genetic model of familial ALS, whereby a delay of ALS symptom onset and an extension of lifespan was observed in male G93A SOD1 mice, whereas females showed improved motor performance. Notably, we also observed sex-dependent effects of REL-1017 on molecular markers of neuronal plasticity and NMDAR subunits. Therefore, also considering the excellent tolerability of REL-1017 demonstrated in clinical trials for major depression (15, 16), this study provides preclinical *in vivo* evidence supporting the clinical evaluation of REL-1017 in ALS.

## Data availability statement

The raw data supporting the conclusions of this article will be made available by the authors, without undue reservation.

## Ethics statement

The animal study was approved by Organismo Preposto al Benessere degli Animali (University of Padova) and Italian Ministry of Health. The study was conducted in accordance with the local legislation and institutional requirements.

## Author contributions

MC: Data curation, Formal analysis, Investigation, Methodology, Validation, Writing – original draft. AS: Data curation, Formal analysis, Investigation, Validation, Writing – original draft. DG: Data curation, Formal analysis, Investigation, Writing – review & editing. HK: Formal analysis, Investigation, Writing – original draft. PM: Conceptualization, Project administration, Writing – review & editing. GM: Conceptualization, Formal analysis, Project administration, Supervision, Writing – review & editing. SM: Data curation, Formal analysis, Funding acquisition, Project administration, Resources, Supervision, Writing – review & editing.
