# Supplementary material for: Sex-dependent effects of the uncompetitive N-methyl-D-aspartate receptor antagonist REL-1017 in G93A-SOD1 amyotrophic lateral sclerosis mice
Source: Front Neurol. 2024 May 3;15:1384829. doi: 10.3389/fneur.2024.1384829 (PMC11100767; doi:10.3389/fneur.2024.1384829)
Supplement: Supplementary file 1 [file Presentation_1.PPTX]

## Slide 1
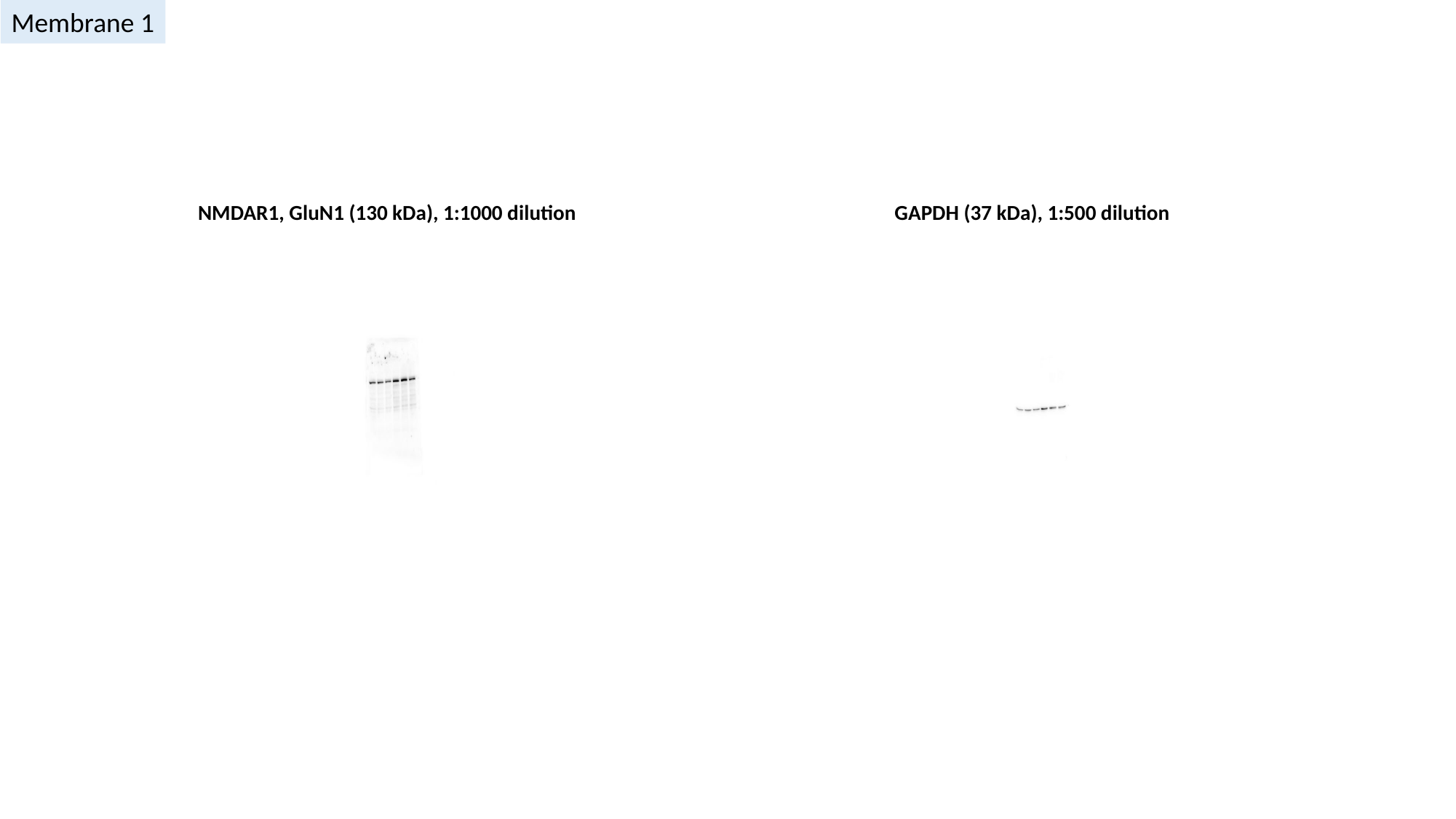

Membrane 1
 NMDAR1, GluN1 (130 kDa), 1:1000 dilution
 GAPDH (37 kDa), 1:500 dilution

## Slide 2
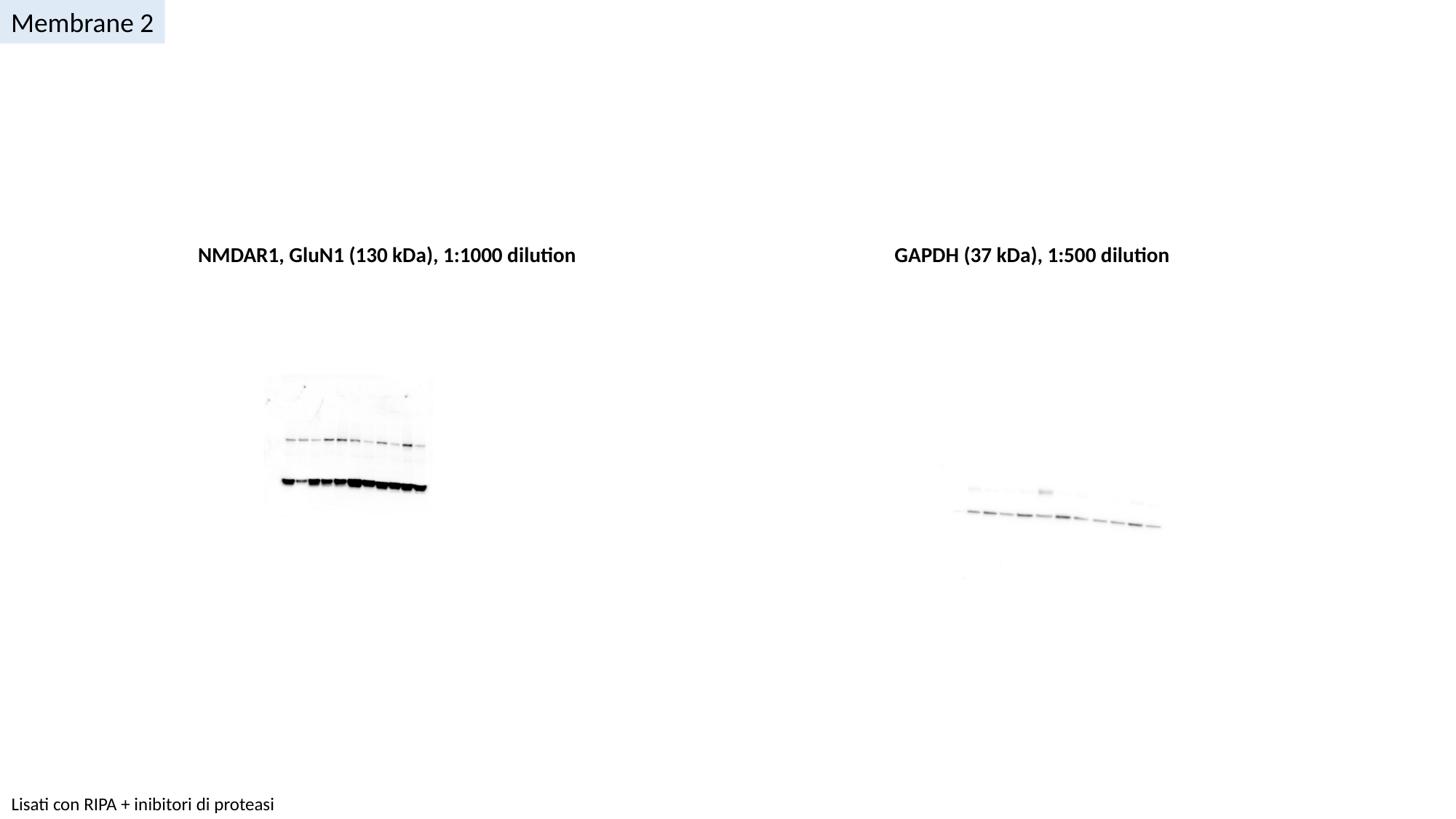

Membrane 2
 NMDAR1, GluN1 (130 kDa), 1:1000 dilution
 GAPDH (37 kDa), 1:500 dilution
Lisati con RIPA + inibitori di proteasi
